# Life histories and ecotype conservation in an adaptive vertebrate: Genetic constitution of piscivorous brown trout covaries with habitat stability

**DOI:** 10.1002/ece3.3828

**Published:** 2018-02-07

**Authors:** Jens Wollebaek, Jan Heggenes, Knut H. Roed

**Affiliations:** ^1^ Department of Natural Sciences and Environmental Health The University College of Southeast Norway Bø i Telemark Norway; ^2^ Department of Basic Science & Aquatic Medicine The Norwegian University of Life Science Oslo Norway

**Keywords:** assortative mating, ecotype, feeding, habitat, life history, piscivory

## Abstract

Ecotype variation in species exhibiting different life history strategies may reflect heritable adaptations to optimize reproductive success, and potential for speciation. Traditionally, ecotypes have, however, been defined by morphometrics and life history characteristics, which may be confounded with individual plasticity. Here, we use the widely distributed and polytypic freshwater fish species brown trout (*Salmo trutta*) as a model to study piscivorous life history and its genetic characteristics in environmentally contrasting habitats; a large lake ecosystem with one major large and stable tributary, and several small tributaries. Data from 550 fish and 13 polymorphic microsatellites (*H*_e_ = 0.67) indicated ecotype‐specific genetic differentiation (θ = 0.0170, *p* < .0001) among Bayesian assigned small riverine resident and large, lake migrating brown trout (>35 cm), but only in the large tributary. In contrast, large trout did not constitute a distinct genetic group in small tributaries, or across riverine sites. Whereas life history data suggest a small, river resident and a large migratory piscivorous ecotype in all studied tributaries, genetic data indicated that a genetically distinct piscivorous ecotype is more likely to evolve in the large and relatively more stable river habitat. In the smaller tributaries, ecotypes apparently resulted from individual plasticity. Whether different life histories and ecotypes result from individual plasticity or define different genetic types, have important consequence for conservation strategies.

## 1 INTRODUCTION

Ecotypes are unique populations that are adapted to their local environment (Turesson, 1922). Adaptations to optimize reproductive success promote different life history strategies (Cole, 1954), but to what extent different life histories evolve into separate gene pools is less known (Pelletier, Garant, & Hendry, 2009). Recognition of sympatric life history variants as genetically structured ecotypes is critical for developing proactive conservation strategies and management (Couturier, Otto, Cote, Luther, & Mahoney, 2010; Foote, Newton, Piertney, Willerslev, & Gilbert, 2009; Segura, Rocha‐Olivares, Flores‐Ramirez, & Rojas‐Bracho, 2006; Wood, Bickham, Nelson, Foote, & Patton, 2008). Environmental characteristics promoting adaptation manifested in genetic structure are fundamental in evolutionary biology, for example, the continuous process of ecological speciation (Hendry, 2009), and in a population conservation perspective to maintain intraspecific diversity (Heywood, 1995).

Freshwater fishes are vertebrates that may be morphologically and genetically variable with considerable genetic structuring related to local adaptation (Carvalho, 1993; Carvalho & Hauser, 1994; Heggenes, Roed, Jorde, & Brabrand, 2009; Koskinen, Haugen, & Primmer, 2002; Seehausen & Wagner, 2014). For example, salmonids display a wide variety of life history strategies (Hendry & Stearns, 2003), presumably often representing sympatric ecotypes (Adams et al., 2016; Goetz et al., 2010; Ramstad, Woody, & Allendorf, 2010; Taylor, 1999). Traditionally, however, ecotypes have been defined based on life history characteristics rather than genetic studies, potentially confounding individual plasticity with genetic structures (Crispo, 2008). In highly exploited species in particular, monitoring neutral or putative adaptive genetic divergence across environmental gradients is critical to establish conservation measures that ensure population survival and evolutionary potential (Baillie, Muir, Hansen, Krueger, & Bentzen, 2016).

The common and geographically widespread brown trout (*Salmo trutta* L.) is a polytypic species (Elliot, 1994) that exhibits a wide range of life history strategies, exemplified by 4‐year‐old wild trout varying in size from 20 to 1 kg (Klemetsen et al., 2003). Body size is a key feature that will influence population structure (Werner & Gilliam, 1984), and species with large variation in size are of particular interest. The causes of size variation in populations are complex, but differentiated resource exploitation (i.e., foraging specializations) could drive population divergence (Foote et al., 2009; Kume, Kitano, Mori, & Shibuya, 2010; Segura et al., 2006; Taylor, 2015; Werner & Gilliam, 1984). Ontogenetic habitat shifts toward environments promoting growth are often found in fish (Northcote, 1978).

Piscivory as part of a life history is required for many fish species like brown trout to grow large (Mittelbach & Persson, 1998). In brown trout, it is common and typically involving migrations, and habitat shifts from riverine recruitment to lacustrine feeding habitat with concomitant shifts in growth (Aass, Nielsen, & Brabrand, 1989; Jonsson, Naesje, Jonsson, Saksgard, & Sandlund, 1999). The timing and extent of piscivory onset vary with community configurations, resulting in distinct shifts within populations (Jensen, Kiljunen, & Amundsen, 2012; Sanchez‐Hernandez, Eloranta, Finstad, & Amundsen, 2017). Empirical evidence for piscivory representing genetically distinct trout ecotypes is, however, generally lacking. Exceptions are unusually large and long‐lived ferox brown trout (Campbell, 1979; Mangel, 1996) and lake trout (*Salvelinus namaycush* W.) (Bernatchez, Laporte, Perrier, Sirois, & Bernatchez, 2016; Marin, Coon, Carson, Debes, & Fraser, 2016; Perreault‐Payette et al., 2017). Ferox trout initially follow the same growth pattern as other brown trout, but shift toward piscivory at total length (*L*_T_) 35–40 cm, resulting in sudden rapid growth as a function of size rather than age (Campbell, 1979). Ferox trout appear to be reproductively isolated (i.e., genetically distinct, from sympatric population pairs of trout both in time and space) (Duguid, Ferguson, & Prodohl, 2006; Ferguson, 2004; Ferguson & Taggart, 1991), spawning earlier in lower and deeper sections of large rivers. Individual‐based models suggest that piscivorous individuals are rare and found when mortality rates are intermediate and littoral volumes are large (Mangel & Abrahams, 2001). Thus, the growth rate required to become piscivorous is only achieved when fish densities and intraspecific competition are restricted. However, survival to an age for sufficient growth to become piscivorous is needed, and the abundance of these piscivorous fish covaries with littoral feeding habitats that favor territorial behavior and growth. Similarly, in lake trout within the highly diverse genus *Salvelinus*, habitat size and depth are positively associated with both growth and longevity (Baillie, Muir, Hansen, et al., 2016; McDermid, Shuter, & Lester, 2010). Thus, selection toward piscivory and large size may be favored in spatially large and presumably stable environments, and retained by size‐assortative mating per se. In contrast, in environmentally unstable habitats, the expression of a phenotype may depend on population density (Simpson, McCaffery, & Hagele, 1999) and optimal life time strategies that vary in time. Large size is often advantageous for survival, but is environmentally dependent (Carlson, Olsen, & Vollestad, 2008; Wilson, Hutchings, & Ferguson, 2003). Disentangling genetic differentiation (i.e., heritable adaptation) from environmental effects (i.e., individual plasticity) in ecotype variation (e.g., size) is challenging and potentially habitat‐specific (Crispo, 2008; de Jong, 2005; Langerhans, 2008). Moreover, empirical evidence exists for increased phenotypic plasticity in fluctuating environments (Niehaus, Wilson, & Franklin, 2006), highlighting the importance of ecotype studies in contrasting environments.

North temperate freshwater fish communities and environments appear to promote sympatric ecotypes, due to depauperate environments, repeated vicariance and dispersal events, environmental heterogeneity, adaptive flexibility of morphological features, and the occurrence of genome duplications (Taylor, 1999). Indeed, the presence of ferox trout correlates with northern oligotrophic waters (Campbell, 1979). Northern alpine low‐density communities thus represent natural systems where piscivorous ecotypes may evolve. We surveyed one such ecosystem with big lakes and several environmentally contrasting tributaries, where consistently rapid growth and large size in piscivorous individuals may indicate genetic fixation of piscivory (L'Abée‐Lund, Aass, & Saegrov, 2002; Tysse, Skaala, & Jenssen, 2004). If present, ecotype variation is an important conservation issue that should be considered when defining management units (Moritz, 1994; Paetkau, 1999), for example, in the context of sport fishing regulation, stocking programs and habitat fragmentation to conserve highly valued and threatened genetic partitions (Allendorf, England, Luikart, Ritchie, & Ryman, 2008; Baillie, Muir, Scribner, Bentzen, & Krueger, 2016; Valiquette, Perrier, Thibault, & Bernatchez, 2014).

Studies of genetic cryptic structures, for example, involving sampling of rare types of highly mobile species in less accessible (i.e., fast flowing and deep) reproduction habitats, bring along logistical and methodological difficulties (Palme, Laikre, & Ryman, 2013). Our study design consequently used wild brown trout in their shared habitat to approximate a common garden experiment in situ. We implemented genetic frequentist assignment and Bayesian clustering approaches to delineate tentative population structures of the piscivorous unit, preselected by life history characteristics to increase potential effect size (Ioannidis, 2005). The objective was to test whether piscivorous large brown trout, a typical life history in many lakes, including the studied ecosystem, also may constitute an ecotype with a genetic signature. Therefore, we test whether brown trout allele frequencies differ between population partitions of typically riverine resident (i.e., small) and large (>35 cm) brown trout within and across environmentally contrasting recruitment and growth habitats.

## 2 MATERIALS AND METHODS

### 2.1 Study site

**Figure 1:**
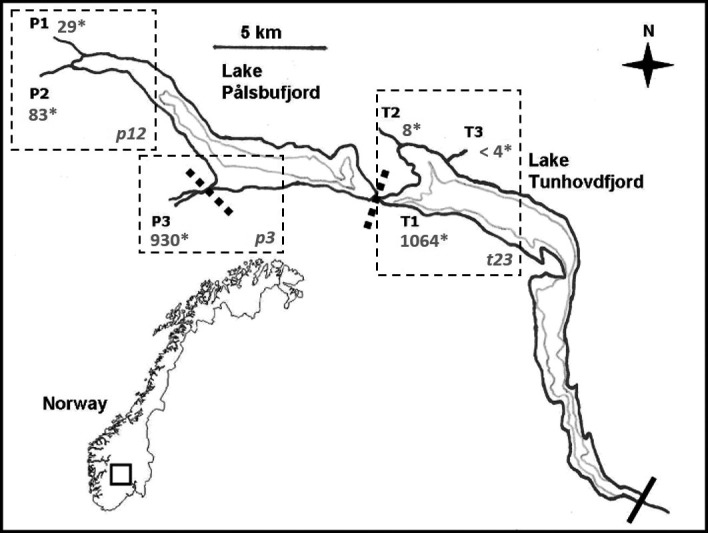
Lake Pålsbufjord and the downstream Lake Tunhovdfjord with sample locations. Minimum water level is indicated by gray line, and the six riverine sites are marked with capital abbreviations. Solid bar indicates semibarrier, broken bars indicate partial restriction to up‐ and downstream migration, and asterisks indicate mean flow (million m^3^ year^−1^). Population clusters p12, p3, and t23, inferred from major STRUCTURE assignment per site are highlighted with dashed rectangles

Lake Tunhovdfjord (TUN) and Lake Pålsbufjord (PAL) are part of a 35 km long sub alpine hydroelectric reservoir, located in south‐central Norway (48°E, 67°N, Figure 1), regulated first in year 1919 by an outflow dam. A hydropower dam separating the two lakes was erected in 1946, restricting the previously free migration between the lakes, to migration through bottom gates in the dam and a 7 m^2^ large and 1.2 km long subterranean anthropogenic channel. Extensive mark–recapture and genetic studies have, however, revealed continued and substantial migration between the lakes (Aass, 1973; Brabrand et al., 2008; Wollebaek, Heggenes, & Roed, 2011). Lake PAL now has a surface area of 5.3–19.5 km^2^ with maximum depth 25 m, 725.5–749.0 m a.s.l. Lake TUN, located immediately downstream, has a surface area of 14.0–25.0 km^2^ and maximum depth 70 m, 718.0–736.0 m a.s.l.

Three contrasting tributaries in each of the two lakes (Figure 1) are potential recruitment areas for the brown trout, making up >99% of available lotic spawning and rearing habitat in the system. In Lake PAL, the largest tributary, River Numedalslågen (P3), has a mean flow (1961–1990) of 930 million m^3^ year^−1^, representing 87% of the total inflow to the lake. The inflow to PAL is an approximately 80 m wide, deep (>2 m) run. Available river habitat for spawning is restricted to the lower 300 m of the river by an upstream waterfall (Figure 1), and about 2,400 m^2^ is available as recruitment habitat. The much smaller River Rambergåi (P2), mean flow (1961–1990) 83 million m^3^ year^−1^, is about 5 m wide and 1.5 km long up to Lake Rambergvatnet (756 m a.s.l.), providing an estimated rearing area of 7500 m^2^. River Halldalsåi (P1), the second small tributary, mean flow (1961–1990) 29 million m^3^ year^−1^, is a 4.5 km long and about 4 m wide stream up to a dam at the outlet of Lake Halldalsvatnet (846 m a.s.l.). It provides an estimated rearing area of 18,000 m^2^. Mean annual water runoff (1987–2007) in two locations within the PALs watershed is 26.7 L s^−1^ m^−2^ (min 17.8, max 36.5, *SD* ± 5.6) and 15.4 L s^−1^ m^−2^ (min 7.3, max 22.9, *SD* ± 3.8), indicating a twofold to threefold variation in annual flow within the alpine watershed. Recruits from all tributaries typically migrate downstream to the common feeding habitat in Lake PAL and TUN at age 2+ or 3+ (Brabrand et al., 2008). In Lake TUN, the former lotic recruitment habitat, also connecting the two lakes, has disappeared because of the dam. Trout now only have an estimated 2,000 m^2^ suitable spawning habitat (at flow 1,064 million m^3^ year^−1^ [1961–1990]) at water depth 5–23 m (T1), immediately downstream the subterranean anthropogenic channel. Available spawning and recruitment habitat size and associated hydraulics fluctuate substantially with water regulations in Lake PAL and TUN. An additional two small tributaries make up the majority of the remaining spawning and recruitment habitat in TUN. In River Rødungselva (T2; mean flow 8 million m^3^ year^−1^ [1961–1990]), upstream migration is restricted by a natural barrier 1.5 km up the about 6 m wide river, resulting in roughly 9,000 m^2^ of available rearing habitat. The small River Tunhovdbekken (T3), mean flow <4 million m^3^ year^−1^ and located close to T2, is 1 km long and about 2 m wide, providing about 2,000 m^2^ of recruitment habitat. Upstream migration access to T3 varies with annual variations in TUN water level.

Brown trout have probably been allopatric native for more than 6,000 years in both lakes (Huitfeldt‐Kaas, 1918; Indrelid, 1985), and sympatric with Arctic char (*Salvelinus alpinus* L.) and European minnow (*Phoxinus phoxinus* L.), both since the two latter were introduced around 1920. No other forage fish species occur, and diets of adult trout in both lakes often consist of terrestrial insects and a variety of zooplankton and zoobenthos. Crustaceans like *Gammarus pulex and Lepidurus arcticus,* known to favor growth, are present in limited amounts (Brabrand et al., 2008; L'Abée‐Lund et al., 2002). Piscivory is now common, with approximately 15% of the adults in TUN in the 1990s being piscivorous. Brown trout in the two lakes may start preying on minnow and char at trout size 15 and 22 cm, respectively (Brabrand et al., 2008; L'Abée‐Lund et al., 2002). Maximum age and weight of wild‐born trout in PAL and TUN exceed 25 years and 15 kg, respectively. It has been thought that mainly the deep habitat in P3 can hold trout of these sizes (Aass, 1973). Fishing pressure on brown trout is low. The annual catch of brown trout in TUN has decreased since the late 1960s and was estimated to 0.3 kg/ha in 1990 (Aass, 1990). Supportive breeding has been implemented since 1970, and approximately 1 million adipose fin‐clipped trout have been released directly in the lakes thereafter. At present, 13,000 1‐year‐old trout are released annually, but mark–recapture and genetic studies indicate lack of reproduction of these stocked fish (Brabrand et al., 2008; Wollebaek, Heggenes, & Røed, 2010; Wollebaek, Roed, Brabrand, & Heggenes, 2012). Progeny of larger fish (>35 cm total length) from these two lakes have also been used for stocking in other reservoirs in Norway, as they were thought to be genetically adapted to fast growth and piscivory (Aass, 1973). Results have revealed that some of these transferred trout become piscivorous and surpass native trout in growth (Aass, 1984).

### 2.2 Sampling

The study complies with the current laws in Norway; ethical concerns on sampling were followed under permission (*2003/9267)* from the County Governor of Buskerud. All efforts were made to ameliorate suffering of animals. Fin clips of 30–40 wild riverine resident trout (R) across year classes were sampled after electroshocking (type FA3, exponential pulses 1,200 V, frequency 86 Hz, made by Geomega a/s, Trondheim, Norway) at each of the six recruitment sites (Figure 1, *n* = 324) during 2005–2007. Samples were collected from the highest regulated water level to 300–800 m upstream, and represent 3‐ to 7‐year classes within sites (age 0+ to 7+, as verified by length measures and aging of a sample), but only age classes 0+ to 2+ in P3. The majority of fish from all sites were nonmature. Wild trout (verified by present adipose fin) from both lakes (L, putative piscivorous and nonpiscivorous ecotypes) were caught annually 2006–2008, by beach seines and gillnets throughout both lakes. Resampled individuals were excluded from further analysis, based on genetic composition (*n* = 14). Sample L consisted of 324 trout (200 trout >350 mm), mean total length (*L*_T_) 404 mm (min 137 mm, max 770 mm, *SD* ± 157), 122 males and 202 females, determined from physical characteristics, gonads and presence of running egg/milt in ripe trout, and across 14‐year classes (age 3+ to 16+). Individual ages of a random sample of riverine resident fish and all lake caught fish were determined by scale and otolith readings, followed by back calculation of age, assuming proportionality among fish length and age structure (Lea, 1910).

### 2.3 Genetic diversity

Genetic variation was analyzed using 13 microsatellite loci (Table A1), following the procedure described in Wollebaek et al. (2010). Temporally resampled trout were identified with CERVUS v.3.0 (Kalinowski, Taper, & Marshall, 2007), and 14 lakes caught trout were excluded based on a full match criteria. Tests for null alleles, large allele dropouts, and scoring errors were performed in MICRO‐CHECKER v.2.2.3 after 10,000 iterations (Van Oosterhout, Hutchinson, Wills, & Shipley, 2004). The program TFPGA v.1.3 (Miller, 1997) was used for descriptive statistics (number of alleles, observed, and expected heterozygosity [*H*_o_ and *H*_e_]). Allelic richness (*A*_r_) among samples was estimated in FSTAT v.2.9.3.2 (Goudet, 1995), and riverine differences in *H*_o_ and *A*_r_ were tested across loci with ANOVA. Possible departure from Hardy–Weinberg (HW) equilibrium for all loci within riverine sites and globally across loci within sites, as well as tests for linkage disequilibrium across sites, were performed in FSTAT, after 104,000 and 780,000 permutations, respectively. The program BOTTLENECK v.1.2.2 (Cornuet & Luikart, 1996) was used to assess possible recolonization or strong genetic drift between age classes within sites, with a Wilcoxon test assuming a two‐phased model with 90% stepwise mutations after 10,000 iterations.

### 2.4 Life history and ecotype variation

Genetic structuring of river and lake caught trout was evaluated with Weir and Cockerham's (1984) pairwise *F*_ST_ (θ) estimate with >15,000 permutations, calculated in FSTAT (Goudet, 1995). Genetic relationships among samples were visualized with PCoA plots of standardized covariance matrixes of genetic distance, estimated in GENALEX v.6.502 (Peakall & Smouse, 2006). Statistical support for observed genetic structures, not possible from PCoA plots, was addressed by bootstrap evaluations of Nei, Maruyama, and Wu's Da (1983) in a neighbor‐joining model after 10,000 iterations, in POPULATIONS v.1.2.3 (Langella, 1999) and TreeView v.1.6.6 (Page, 1996).

Ecotype constitution was tested by comparing pairwise *F*_ST_ between partitions of riverine resident trout and lake caught trout >35 cm assigning to rivers/populations, as stomach analyses generally indicate an upper size limit of approximately 35 cm for nonpiscivorous fish in these lakes (Aass, 1990; Brabrand et al., 2008; L'Abée‐Lund et al., 2002).

Two common assignment procedures for testing the hypothesis were implemented. A frequentist approach first compared all riverine resident fish from sample sites to putative piscivorous trout (*L*_T_ > 35 cm) that assigned to the riverine sites in GENECLASS2 (Piry et al., 2004) with default settings of the frequencies‐based sampling algorithm of Paetkau, Calvert, Stirling, and Strobeck (1995). Straying will decrease self‐assignment of river samples and may cause ecotype hybridization that decreases the power of ecotype constitution tests. Moreover, river sites as a baseline for assignment of lake caught trout will be less adequate if genetic population structure contrasts with geographic structure, for example, in a meta‐population structure where fluctuating niche opportunities determine the extent of population admixture (Hanski & Gilpin, 1997; Wood et al., 2008). First‐generation immigration rates for R‐partitions were therefore calculated from individuals self‐assignment in GENECLASS2 with above settings, and thereafter in the Bayesian STRUCTURE v.2.2 (Falush, Stephens, & Pritchard, 2003; Pritchard, Stephens, & Donnelly, 2000) after 200,000 replications of burn‐in, 500,000 MCMC replicates, population information, and 0.05 prior migration rate. Individuals were treated as migrants in both the frequentist and Bayesian approach when they assigned strongest to a site where it had not been sampled, in GENECLASS2 only when self‐assignment was below 5%.

Migration estimates corroborated previous observations of high straying among sites and a tripartite structure (Wollebaek et al., 2012) that may bias assignment of lake caught trout and interpretation of genetic partitions based on river sites. Bayesian clustering in STRUCTURE was accordingly used to infer a more biological relevant population composition in samples pooled (including small‐sized trout caught in the lake to maximize Bayesian power), and within riverine sites, using 10 iterations of 1–15 population clusters (*K*), 200,000 replications of burn‐in, 500,000 MCMC replicates, and admixture model with correlated allele frequencies. The number of population clusters (*K*) was estimated by maximum‐likelihood measures (Ln P(*D*)) and their variance and by Δ*K* (Evanno, Regnaut, & Goudet, 2005). All trout (*n* = 550) were assigned to riverine (R) and lake (L) partitions of population clusters identified by STRUCTURE, based on individual membership coefficients (*q*). Population hybrids as in the studied lakes (Wollebaek et al., 2012) and ecotype hybrids, both potentially characterized by low *q*‐values, may represent temporary components of long‐term population units (Edmands, 2007), which reduce power of testing for population structures. The power for testing for piscivory as a genetically defined ecotype also increases with effect size, that is, comparing contrasting size subsamples. For hypothesis testing, we define tentative ecotype populations following size (*L*_T_ > 35 cm) and assignment criteria. A stringency level of *q* = 0.8 from the STRUCTURE analyses was implemented in assigning individuals, excluding trout with lower assignment from populations. The conservative low‐assignment threshold (Vaha & Primmer, 2006) was preferred since this and earlier studies (Wollebaek et al., 2010, 2012) indicated considerable straying and hybridization among riverine sites, and since the sample covered 16 years (back‐calculated age) with possible drift among year classes, not covering the entire available habitat within streams. Based on documented positive covariation between size and extent of piscivory, ecotype testing was performed on length‐defined subsamples of lake caught fish. Genetic relationship among partitions was visualized using PCoA and Nei et al.'s Da (above). A hierarchical AMOVA was used to quantify the allelic variance of R and L within and across populations in ARLEQUIN v.3.1 (Excoffier, Laval, & Schneider, 2005).

The sequential Bonferroni correction procedure (Rice, 1989) was used to reduce Type 1 errors for multiple genetic tests. ANOVA and Tukey–Kramer HSD compared means of length (*L*_T_) and assignment (*q*) of population partitions in R v.3.3.2 (http://www.R-project.org), only considering comparisons of samples with *n* ≥ 5. Tests implementing all sizes (*L*_T_ > 13.7 cm) and *L*_T_ of 40–50 cm in the lake sample L evaluated the robustness of the defined size limit for *F*_ST_ differentiation in the Bayesian design. The suitability of the implemented threshold for assignment in this design was tested by running additional *F*_ST_ tests implementing *q*‐levels from 0.7 to 0.9.

## 3 RESULTS

### 3.1 Genetic composition

Amplification and allele calling were obtained in 99.2% of the cases, and consistent with secondary amplification, and controls. Number of alleles per locus averaged 14.1 (range 2–35, *SD* ± 11.7, Table A1). Quality control screening did not reveal indications of scoring error due to stuttering, large allele dropouts, or null alleles. After correction for multiple tests, no deviations from HW were found within recruitment sites (*p* > .012 within loci, *p* > .346 across loci), and nonrandom associations of alleles among loci were not found across samples (*p* > .0008). No significant differentiation in *H*_e_ or *A*_r_ was found among riverine sites (Table A1, ANOVA, *F* < 0.495, *p* > .779). The test for bottlenecks within sites did not indicate recolonization or strong genetic drift between age classes (*p* > .271).

### 3.2 Genetic structure

**Figure 2:**
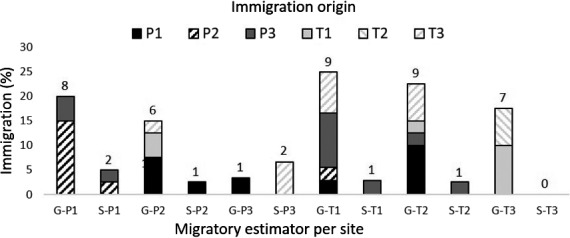
Migration among riverine sites. First‐generation (F0) immigrants to riverine sites estimated in GENECLASS2 (G) and STRUCTURE (S). Proportion of dispersed fish (%) are divided into source of origin (six sites, total *n* = 226, total number dispersed within sites on top of bars)

**Figure 3:**
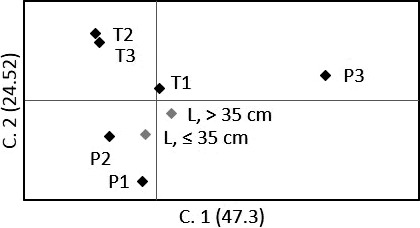
Principal coordinates analysis of geographic samples. Multivariate variation among trout (*n* = 550) from river sites (P/T in black) and lakes (L in gray), from standardized covariance matrixes of genetic distance

**Figure A1:**
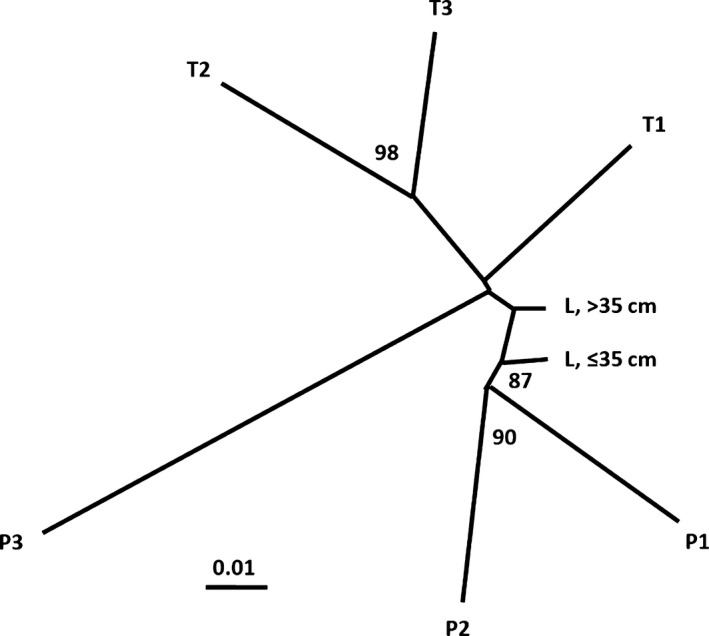
Neighbor‐joining tree of all trout (*n* = 550) from river sites (P/T) and lakes (L). Based on Nei et al.'s (1983) genetic distance Da. Bootstrap support based on 10,000 replicates is shown when >50. See section 2.2 and Table 1 for sample sizes

Allele frequencies of all riverine sites were significantly differentiated (mean θ: 0.0332, *SD* ± 0.0184, *p* < .0001, Table A2). Lake caught trout assigned to the six riverine sites in GENECLASS2 were differentiated similarly (mean θ: 0.0211, *SD* ± 0.0089, *p* < .0089). Pairwise *F*_ST_ estimates were somewhat larger among R and L in P3 (θ: 0.0088, *p* = .0429), compared to other sites (mean θ: 0.0027, *SD* ± 0.0022, *p* > .1501), but did not indicate ecotype variation within any of the six sites after correction for multiple tests (Table A2). The frequentist assignment however, only correctly traced 64% (P1:73, P2:60, P3:87, T1:44, T2:53, T3:70) of the riverine resident fish to their sampled site. Estimates of straying varied considerably among sites and approach, from 7 to 40 first‐generation migrants in total, in STRUCTURE and GENECLASS2, respectively (Figure 2). A superior tripartite genetic structure was supported by PCoA plots and estimated genetic distances (Da) among sampled sites, that is, forming only three clades (Figure 3; 71.8% of the total variation explained by the first two components, Figure A1; >90% bootstrap support for clustering of geographically close rivers within lakes). This potentially confounded sitewise interpretation of genetic partitioning, but fitted overall biological expectations.

**Figure 4:**
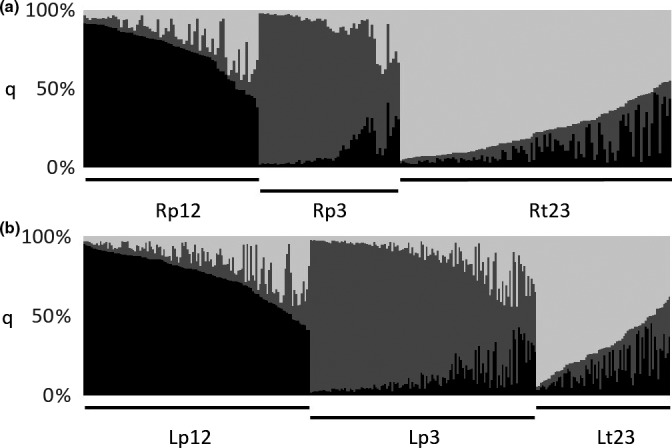
Population clusters. Individual membership coefficients (*q*) of riverine resident trout (a, *n* = 226) and lake caught trout (b, *n* = 324) divided into three population clusters (p12: black, p3: dark gray, t23: light gray) without prior information in STRUCTURE

**Figure A2:**
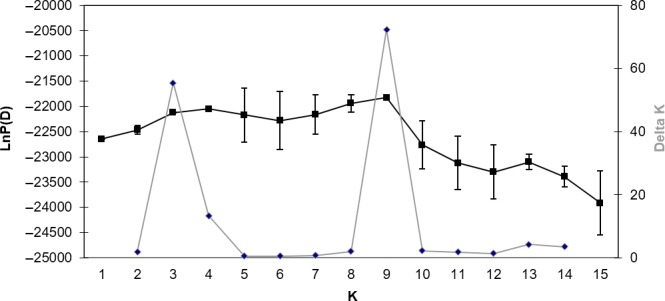
Bayesian support for a tripartite structure. Mean posterior probability Ln P(*D*) with standard deviance (bars) and delta *K*, after 10 runs for *K* population clusters in STRUCTURE. The analysis is for all fish (*n* = 550), without prior sample information and with an admixture model

**Figure A3:**
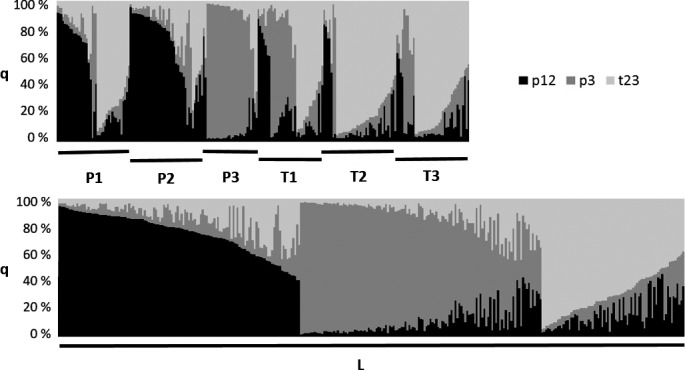
Individual membership coefficients of the tripartite structure. Bars indicate individual membership coefficients, from river sites (P/T, *n* = 226) and lakes (L, *n* = 324), of the three cluster populations (*K* = 3), denoted by the three colors

STRUCTURE analyses provided strongest support for a tripartite partition, considering both the diminishing probability of *K* > 3 and the estimated Δ*K* (Figure A2). A main structure of *K* = 3 was assumed despite a somewhat larger Δ*K* for nine population clusters, as the model used in STRUCTURE easily overestimates *K* (Falush et al., 2003), and as Δ*K* = 9 was highly dependent on reduced Ln P(*D*) for *K* = 10. The population clusters were named p12, t23, and p3, named according to their most likely origin. Clusters p12 and t23 corresponded mainly to the two smaller tributaries in PAL (P1 and P2) and the small tributaries in TUN (T2 and T3), respectively, whereas population cluster p3 corresponded to the large river (P3) in PAL. Sample T1 and L had a more mixed genetic origin, with the latter assigning slightly less to the population cluster corresponding to the small tributaries in TUN (Table 1, Figure A3). Sample L assigned equally in numbers to the three population clusters in all three years (data not shown). STRUCTURE analyses did not identify any substructure within river samples. Population clusters include riverine (R) and lake (L) partitions (Figure 4). Following the Bayesian assignment‐ and size criteria, these are referred to as partitions within populations (e.g., riverine sample of population cluster p12 is named Rp12).

**Table 1:** Sample contributions to the three population clusters, estimated without prior population information in STRUCTURE. Cluster assignment (highest *q*) marked with asterisks

Sample site	*n*	Population cluster		
		p12	p3	t23
P1	40	0.461*	0.124	0.415
P2	40	0.664*	0.127	0.209
P3	30	0.105	0.767*	0.128
T1	36	0.253	0.366	0.381*
T2	40	0.166	0.132	0.701*
T3	40	0.162	0.205	0.633*
L	324	0.373*	0.364	0.263

### 3.3 Population and ecotype partition

**Figure A4:**
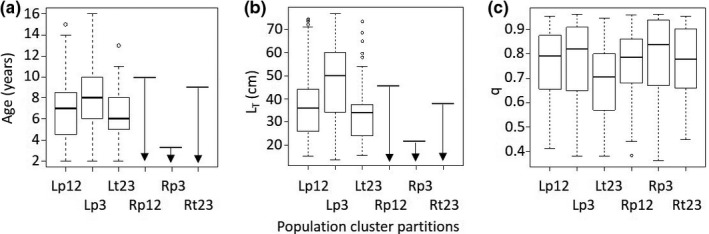
Age, length, and assignment variation among population cluster partitions. Tukey box plots of age (a, years), total length (b, *L*_T_), and assignment (c, *q*) of all lake caught (L, *n* = 324) and riverine resident (R, *n* = 226) partitions of population clusters. Only maximum age and length of riverine resident fish are measured, and thus presented

Mean size (*L*_T_) of lake caught trout differed among population clusters (*p* < .001, Figure A4) and were considerably larger in Lp3 (mean 470, *SD* ± 165), than in Lp12 (mean 377, *SD* ± 145) and Lt23 (mean 339, *SD* ± 121). Pairwise comparisons indicated no size difference between Lp12 and Lt23 (*p* = .198). Ages were similarly differentiated (*p* < .001) among population clusters, considerably larger in Lp3 (mean 7.94, *SD* ± 2.92), but no difference (*p* = .536) between Lp12 (mean 6.72, *SD* ± 2.79) and Lt23 (mean 6.30, *SD* ± 2.25)) (Figure A4). Self‐assignments (*q*) were highest in the two ecotype partitions in p3 (Figure A4) and differed among population cluster partitions in total (*p* < .013). With respect to ecotype partitioning within population clusters, *q* only differed among partitions in t23 (*p* = .020).

**Figure 5:**
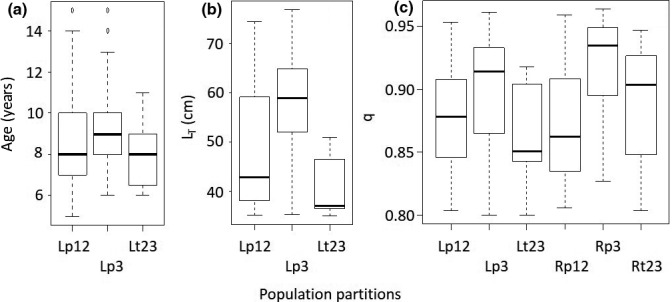
Age, length, and assignment variation among population partitions. Tukey box plots of age (a, years), total length (b, *L*_T_), and assignment (c, *q*) of lake caught (L, *n* = 95) and riverine resident (R, *n* = 113) partitions of populations, post exclusion of lake caught fish ≤35 cm and fish with assumed hybrid origin (*q* < 0.8)

The two exclusion criteria (*L*_T_ ≤ 35 cm and *q* < 0.8), respectively, excluded 124 and 295 trout, combined retaining 208 trout across population partitions (Table 2). Lake caught trout size still differed among populations (*p* < .001, Figure 5) and were considerably larger in Lp3 (mean 576, *SD* ± 109), than in Lp12 (mean 481, *SD* ± 131) and Lt23 (mean 409, *SD* ± 63). Pairwise comparisons indicated similar size (*p* = .185) in Lp12 and Lt23. Ages were less differentiated for this subsample (*p* = .059 among populations, still largest in Lp3, Figure 5). Population self‐assignments (*q*) were still highest in the two ecotype partitions in p3 and differed among population partitions in total (*p* < .001, Figure 5). No differences in *q* were found among partitions within populations (*p* > .355).

**Table 2:** Weir and Cockerham's (1984) pairwise *F*_ST_ (θ) estimate among river resident (R, *n* = 113) and large (L, >35 cm, *n* = 95) lake caught trout above the diagonal and among riverine resident (R, *n* = 113) and small (L, ≤35 cm, *n* = 47) lake caught trout below the diagonal, all assigned (*q* > 0.8) to STRUCTURE population clusters p12, p3, and t23. Nonsignificant (ns) and significant tests (*, **, ***) after sequential Bonferroni correction according to α = 5%, 1%, and 0.1%, respectively

Population partition (*n*)	Rp12	Rp3	Rt23	Lp12	Lp3	Lt23
Rp12 (33)		0.0748***	0.0419***	0.0019 ^ns^	0.0482***	0.0383***
Rp3 (29)	0.0748***		0.0897***	0.0853***	0.0170***	0.0802***
Rt23 (51)	0.0419***	0.0897***		0.0442***	0.0665***	0.0064 ^ns^
Lp12 (34/23)	−0.0010 ^ns^	0.0760***	0.0492***		0.0495***	0.0335***
Lp3 (50/14)	0.0670***	0.0312 ^ns^	0.0808***	0.0631***		0.0567***
Lt23 (11/10)	0.0385***	0.0994***	−0.0036 ^ns^	0.0409***	0.0767***	

**Figure 6:**
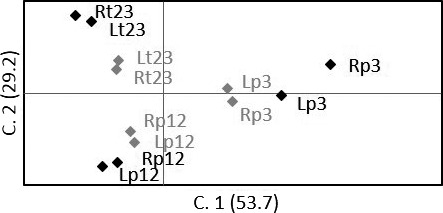
Genetic distance of population partitions. Multivariate variation (principal coordinates analysis) among all riverine resident (R) and lake caught trout (L) assigned to STRUCTURE population clusters (p12, p3, and t23). Markers in black represent population partitions after exclusion criteria (*q* > 0.8, *L*_T_ lake caught trout >35 cm, *n* = 208), and markers in gray represent individuals excluded by these criteria (*n* = 342)

**Figure A5:**
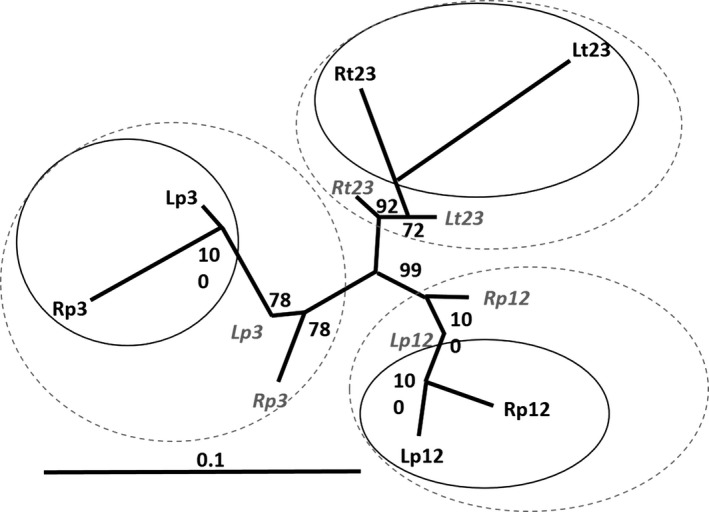
Genetic distance of partitions. Neighbor‐joining tree of all riverine resident (R) and lake caught trout (L) assigned to STRUCTURE population clusters (p12, p3, and t23, gray‐dashed circles), and to populations after exclusion criteria (*q* > 0.8, *L*_T_ lake caught trout >35 cm, black solid circles). Based on Nei et al.'s (1983) genetic distance Da, with bootstrap support based on 10,000 replicates shown when >50

Pairwise *F*_ST_ estimates indicated that ecotype large lake caught trout (>35 cm) were significantly different from ecotype riverine resident trout in population p3 (Table 2, θ = 0.017, *p* < .001), that is, in the large tributary P3. In contrast, lake and river caught trout were genetically similar in the two other populations (θ < 0.006, *p* > .054), corresponding to the smaller tributaries. Consequently, PCoA plots of clusters with subsets of population partitions based on size‐ and assignment criteria, where 82.9% of the genetic variation was captured by the first two components, visualized contrasting ecotypes in p3 only (Figure 6). Furthermore, testing of riverine residents and small lake caught trout (≤35 cm) did not indicate any genetically differentiated ecotypes in any populations (Table 2, θ < 0.031, *p* > .029). Lake caught trout were genetically different across populations for both large (>35 cm, θ > 0.034, *p* < .001) and small (≤35 cm, θ > 0.041, *p* < .001) fish, corroborating the absence of evidence for a common piscivorous ecotype across populations, as indicated by PCoA plots (Figure 6), estimated Da (Figure A5), and the Bayesian tripartite structure without a superior large trout cluster.

AMOVA results attributed the majority of allelic variation to individuals (94.1%, *p* < .001), and greater, but not significant variance to differences among ecotype partitions across the tripartite population structure (5.1%, *p* = .069) than among R and L within populations (0.8%, *p* = .001). Excluding populations one by one only changed the pattern of low but significant variation among partitions within populations when removing p3 (0.3%, *p* = .150), suggesting the significant difference among R and L was caused by population p3‐specific ecotype differentiation.

Implementing size limits of 40, 45, and 50 cm for lake caught trout did not change the pattern of size‐ or assignment differentiation among population partitions (data not shown). Gene frequencies of partitions within populations were also consistently differentiated in p3 (θ > 0.016, *p* < .001), but not within other populations, for these size limits. *F*_ST_ test implementing *q*‐level 0.7 indicated partition differentiation in p3 and t23 (θ > 0.007, *p* < .015), whereas a *q*‐level of 0.9 only differentiated partitions in p3 (θ = 0.021, *p* < .001). Thus, the exclusion criteria of *q* = 0.8 seemed appropriate for hypothesis testing in this system, characterized by high straying rates and occasionally environmental obstacles for spawning migration, as often evident in northern alpine communities of piscivorous trout.

## 4 DISCUSSION

The present study indicated that, in stable environments, large piscivorous trout may be genetically distinct from generally smaller nonpiscivorous conspecifics. Frequentist and Bayesian clustering approaches differed somewhat in their feasibility to test the hypothesis regarding genetically defined ecotype variation. The frequentist approach first suggested more differentiation between population partitions in large stable habitat, but this result was not significant after sequential Bonferroni correction. The Bayesian approach, however, with its increased power of detecting temporal relevant population structure, documents that large piscivorous trout were significantly genetically differentiated from smaller riverine resident trout within one of the studied populations, that is, the large tributary population. The analysis did not, however, find evidence for common piscivorous trout ecotypes with a genetic signature across populations. Therefore, results indicate that the genetically distinct piscivorous ecotype has evolved within the one river with large water flow and environmentally stable habitat, but not within the smaller tributaries providing less and less stable habitat.

Life history strategies manifested as ecotypes are fundamental in evolutionary biology (Turesson, 1922). The diversity of fish life histories has received special interest (Ciannelli, Bailey, & Olsen, 2015; Mehner, Freyhof, & Reichard, 2011; Taylor, 1999). Genetically distinct ecotypes of fish are often documented between migratory and resident life histories (Lin, Quinn, Hilborn, & Hauser, 2008; Pearse et al., 2009; Taylor, 1999), but only sparsely so in brown trout, possibly in part as a result of sample bias (Hindar, Jonsson, Ryman, & Stahl, 1991; Krueger & May, 1987; Skaala & Naevdal, 1989). As in the present investigation, an ecotype signal may be less pronounced than genetic differentiation among geographically defined populations, and studies may give ambiguous results, often related to the different populations studied (Bernatchez et al., 2016; Docker & Heath, 2003; Heath, Bettles, Jamieson, Stasiak, & Docker, 2008; Marin et al., 2016; Olsen, Wuttig, Fleming, Kretschmer, & Wenburg, 2006; Wilson et al., 2008). Sympatric populations with different life history strategies are typically more similar to each other, than to geographically separated populations (Aykanat et al., 2015; Bernatchez et al., 2016; Docker & Heath, 2003; Hindar et al., 1991; Narum, Contor, Talbot, & Powell, 2004; Perreault‐Payette et al., 2017). Therefore, some a posteriori approaches may be insufficient for empirical identification of cryptic genetic structures needed for conservation measures (Palme et al., 2013). The present study demonstrated the increased power of combined genetic approaches to detect weak but biological relevant structures. Designing and implementing studies of tentative populations also across environmentally contrasting habitats and population segments, as in our study, are likely to help unveil possible habitat‐induced ecotype variation.

Although piscivorous life histories are found across a range of taxa (Mittelbach & Persson, 1998), we are aware of few studies comparable to the present where piscivory potentially manifested as a genetically distinct ecotype has been investigated (Bernatchez et al., 2016; Duguid et al., 2006; Ferguson & Taggart, 1991; Perreault‐Payette et al., 2017). This is surprising, considering the ecological importance of top predators in aquatic ecosystems and the structuring of fish communities (Jackson, Peres‐Neto, & Olden, 2001; Kitchell, Eby, He, Schindler, & Wright, 1994). The previous studies of ferox trout considered delayed sexual maturation and longevity as prerequisites for evolution of piscivorous populations. They did not find evidence for a piscivorous ecotype across populations, although the authors stated that this was likely. However, they did find an association between genetically defined large ferox trout and large river habitats, and possible evolution of spatially and temporally separated size‐based sympatric ecotypes within lakes (Duguid et al., 2006). Moreover, using a subset of SNP‐markers, Bernatchez et al. (2016) demonstrated the possible contemporary evolution of both sympatric and allopatric piscivorous lake trout.

Interpretation of ecotype variation may be confounded by random effects of isolation by distance, similar to homing in salmonids (Quinn & Tallman, 1987; Stuart, 1957) which may cause population differentiation even within tributaries as a function of distance (Carlsson, Olsen, Nilsson, Overli, & Stabell, 1999; Vaha, Erkinaro, Niemela, & Primmer, 2007), rather than adaptation. Here, we designed and implemented a study that demonstrated ecotypes have evolved within the large, but short (300 m) river representing a restricted spawning and recruitment habitat area, thus most likely reflecting ecological and behavioral interactions constituting ecotype variation in close proximity.

Random genetic drift is unlikely as an explanatory factor for the observed differentiation of riverine resident and piscivorous trout, due to high longevity and iteroparity, and no more than two generations separate the samples of the two ecotypes. Genetic partition in brown trout is thought to be relatively stable across decades (Palme et al., 2013). Neither is population replacement by neighboring populations likely, as indicated by the lack of bottleneck signals. Meta‐population structures are more likely to occur in small unstable tributaries (Ostergaard, Hansen, Loeschcke, & Nielsen, 2003), and riverine resident trout in the large tributary (P3) were significantly different from all other populations. Founding effects caused by stocking may be excluded as only supportive breeding is implemented. Besides, previous studies indicate no or only marginal reproduction in stocked fish (Wollebaek et al., 2010, 2012). The aforementioned studies of ferox trout and lake trout attributed the genetic differentiation to reproductive isolation both in time and space (Duguid et al., 2006; Ferguson, 2004; Ferguson & Taggart, 1991) and presence of large and deep habitat with the associated potential for spatial separation (Baillie, Muir, Hansen, et al., 2016; Mangel & Abrahams, 2001; McDermid et al., 2010). In our study, large trout in the large tributary (P3) also spawn around the same time as trout in the other tributaries, and temporal variation in spawning (Aykanat et al., 2015; Gharrett, Joyce, & Smoker, 2013), as Duguid et al. (2006) found, is not known. Divergent habitat use by trout with different life histories (McLaughlin, Ferguson, & Noakes, 1999; Morinville & Rasmussen, 2006) or limited sample sizes, inadvertently resulting in selective sampling, could explain why differentiated ecotypes were found only for the largest site here, despite a considerable number of large trout assigning to the smaller tributaries. Whereas trout were sampled across the entire habitat width in the smaller tributaries, electroshocking was feasible only along the stream banks for the large river. Thus, ecotype variation may remain hidden within the smaller sites studied, where the riverine sample possibly constitutes a mixture of both ecotypes. Hence, detecting ecotype variation may be sensitive to both sample locations and size (Palme et al., 2013). A possible alternative scenario is that the large river sample in P3 primarily represents a resident, as opposed to a nonpiscivorous, ecotype. However, the total absence of larger trout caught by electroshocking in the large river P3 weakens this hypothesis. Thus, we find it likely that the two ecotypes observed represent alternative feeding strategies.

The riverine habitat of the piscivorous ecotype in P3 differs from the other sites. River size (including water flow and depth) is large, and river length is short. Although several exceptions exist, fish are generally larger bodied in large and fast flowing rivers (Langerhans, 2008; Quinn, 2005), and body length of returning salmonids may increase with both water discharge and spawning migration length (Power, 1981; Schaffer & Elson, 1975), possibly more so for discharge (Jonsson, Hansen, & Jonsson, 1991). Genetically, distinct ferox trout spawn in lower and deeper sections of large rivers (Duguid et al., 2006; Ferguson, 2004; Ferguson & Taggart, 1991), and large brown trout typically utilize fast and deep water with coarser substrate for spawning (Ottaway, Carling, Clarke, & Reader, 1981; Wollebaek, Thue, & Heggenes, 2008). Theoretical models also predict that water flow influences phenotypic outcomes (i.e., piscivorous phenotypes). Experiments by Keeley, Parkinson, and Taylor (2007) indicated that the majority of morphological variation in rainbow trout (*Oncorhynchus mykiss* W.) was explained by ecotypes (i.e., piscivory), to a minor extent also water flow. Thus, evolution of large piscivorous ecotypes is most likely positively correlated with water flow. Longevity may also induce self‐energizing effects of ecotype differentiation. Achieved length and age of trout in our studied populations equal or even surpass that from other populations defined as large piscivorous brown trout (Campbell, 1979; Jensen et al., 2008; Mangel, 1996). Selection for size is reinforced because females prefer mating with even larger males (Labonne et al., 2009), promoting assortative mating in suitable habitat, that is, larger fish require larger habitats. Although the low‐genetic differentiation among ecotypes in P3 indicates that the reproductive barrier is not absolute, alternative reproductive strategies (i.e., sneaking, Avise, Jones, Walker, & DeWoody, 2002), likely have limited impact on genetic structure. Thus, reproductive isolation may be size‐ and habitat‐specific (Perez‐Figueroa, Cruz, Carvajal‐Rodriguez, Rolan‐Alvarez, & Caballero, 2005), supporting the observed modest gene flow among the sympatric ecotypes in the particularly large and high flow P3 habitat. In this habitat only, long‐term survival of ecotype hybrids, even among small piscivorous and large nonpiscivorous individuals, seems to be limited. Also, in this habitat, both sympatric ecotypes seem to be genetically resilient to gene flow from other tributaries, despite a long history of supportive breeding and a considerable straying among tributaries. We may further speculate that the short spawning run distance additionally increases postspawning survival, iteroparity rates and longevity, favoring evolution of a large‐sized piscivorous ecotype given high water flow.

In contrast, in smaller habitats selection for large size, as a reproductive strategy may be traded off against harsh climatic conditions (Borgstrom & Museth, 2005; Crecco & Savoy, 1985) and predator dilution effects (Brannas, 1995). Carlson et al. (2008) reviewed size dependency of survival, finding that bigger is not always better. Both phenotypic plasticity and genetic variance may increase as a response to fluctuating selection (Crispo, 2008; Rueffler, Van Dooren, Leimar, & Abrams, 2006). In the smaller tributaries, our results support the bet‐hedging theory, suggesting that variation in size is higher in unpredictable environment (Marshall, Bonduriansky, & Bussiere, 2008; Olofsson, Ripa, & Jonzen, 2009). Relatively, large trout will only have a fitness advantage if homing and spawning are in sufficient water volumes and flows. Life history plasticity (i.e., growth and reproduction) in less stable environments (Langerhans, 2008) is also supported by the increased genetic diversity among the smaller tributaries, and higher migration rates among these sites (Wollebaek et al., 2010; this study [*F*_ST_]) may hamper evolution of locally adapted habitat specialists (Crispo, 2008; Sultan & Spencer, 2002).

## 5 CONCLUSIONS

The study demonstrated genetic differentiation of large individuals in a large, stable river habitat. A piscivorous life history did not, however, involve a genetic signature within more unstable habitats or across populations. Rather, it results from individual plasticity. Available river habitat for spawning and recruitment varied considerably among sites, and also indicated a larger proportion of large piscivorous trout in stable habitat. The study corroborates the accumulating evidence that genetic constitution and heritability of life history traits are influenced by environmental stability (Charmantier & Garant, 2005; Sgro & Hoffmann, 2004). Environmental gradients may retain ecotype variation (Perez‐Figueroa et al., 2005), and adaptations to divergent habitat use may cause ecotype variation as a by‐product of divergence (Kume et al., 2010). Conservation of ecotypes to ensure evolutionary potential of populations should therefore rest upon conserving key habitats. Large rivers, regardless of their length, may be key habitats for genetically distinct piscivorous trout. Moreover, piscivorous ecotypes may contribute to population genetic structure and may be a valuable evolutionary resource for future management and supportive breeding. Further studies that assess population dynamics, life history traits, and genomics of piscivory across environmental gradients are warranted to address evolution and conservation of intraspecific diversity.

## CONFLICT OF INTEREST

None declared.

## AUTHOR CONTRIBUTIONS

All authors; JW, JH, and KH conceived the study and participated in the project design. JW conducted the field and laboratory work, and did the genetic analyses. All authors wrote and approved the final manuscript.

## APPENDIX 1

### 1.1

**Table A1:** Sample locations with number of trout, number of amplified individuals (*N*), number of alleles (*N*_all_), expected and observed (direct count) heterozygosity (*H*_o_ and *H*_e_), and allelic richness (*A*_r_)

Loci	P1 (*N* = 40)					P2 (*N* = 40)					P3 (*N* = 30)				
	*N*	*N*_all_	*H*_e_	*H*_o_	*A*_r_	*N*	*N*_all_	*H*_e_	*H*_o_	*A*_r_	*N*	*N*_all_	*H*_e_	*H*_o_	*A*_r_
Strutta12	40	14	0.8003	0.7500	12.1	40	14	0.8916	0.7750	13.0	28	9	0.7793	0.8214	9.0
Str15	40	6	0.7678	0.8250	6.0	40	6	0.7316	0.7750	5.7	30	5	0.7111	0.6333	5.0
Strutta58	38	14	0.8584	0.8421	12.6	39	13	0.8882	0.9487	12.4	30	10	0.7628	0.8333	9.9
Str60	40	2	0.3622	0.4250	2.0	40	2	0.4688	0.4000	2.0	30	2	0.4800	0.3333	2.0
Bru13	39	13	0.8757	0.9487	11.9	40	10	0.8653	0.8750	9.7	28	7	0.7092	0.7500	7.0
Bru14	40	4	0.4141	0.4750	4.0	40	4	0.4566	0.5500	3.9	30	4	0.2139	0.1667	3.9
Bru22	39	2	0.3935	0.2821	2.0	40	2	0.3200	0.3500	2.0	28	2	0.3922	0.4643	2.0
Bru25	40	16	0.8616	0.8250	14.1	40	16	0.8847	0.9250	14.9	30	12	0.8589	0.9000	11.8
Bru07	40	7	0.7469	0.8500	6.6	40	6	0.8075	0.8000	6.0	30	5	0.7428	0.8667	5.0
Bru09	35	13	0.6020	0.5714	11.7	39	16	0.7768	0.7436	13.9	28	5	0.4943	0.6429	5.0
Ssosl417	40	8	0.8272	0.8500	7.7	40	9	0.8256	0.8750*	8.9	30	7	0.7050	0.7333	6.9
Ssosl438	39	3	0.4615	0.4103	3.0	40	4	0.3569	0.3500	3.7	30	3	0.1850	0.2000	3.0
Str73	40	3	0.5666	0.5750	3.0	40	3	0.4928	0.6750*	2.9	30	3	0.4861	0.4667	3.0
Average	39.2	8.1	0.6568	0.6638	7.4	39.8	8.1	0.6743	0.6956	7.6	29.4	5.7	0.5785	0.6009	5.7

Loci	T1 (*N* = 36)					T2 (*N* = 40)					T3 (*N* = 40)				
	*N*	*N*_all_	*H*_e_	*H*_o_	*A*_r_	*N*	*N*_all_	*H*_e_	*H*_o_	*A*_r_	*N*	*N*_all_	*H*_e_	*H*_o_	*A*_r_
Strutta12	36	15	0.8789	0.9167	13.8	40	14	0.8119	0.8250	13.2	39	17	0.8606	0.8974	15.2
Str15	36	5	0.7681	0.8889	5.0	40	6	0.7856	0.8250	6.0	39	5	0.7341	0.7692	5.0
Strutta58	36	17	0.8931	0.9167	15.9	39	15	0.8652	0.8718	13.4	40	11	0.8547	0.9000	10.3
Str60	36	3	0.3353	0.2778	2.8	40	2	0.3487	0.4000	2.0	40	3	0.3809	0.3500	2.7
Bru13	34	11	0.8456	0.8824	11.0	39	13	0.7844	0.8205	11.6	40	12	0.8578	0.9250	11.4
Bru14	36	4	0.2724	0.3056	3.8	40	4	0.4038	0.4000	3.9	40	4	0.3194	0.2750	3.7
Bru22	34	2	0.2297	0.2059	2.0	39	2	0.2778	0.2821	2.0	40	2	0.1172	0.1250	2.0
Bru25	36	17	0.9028	0.8333	15.8	40	17	0.8600	0.8500	15.2	40	15	0.8800	0.8750	14.2
Bru07	36	7	0.7388	0.8333	6.9	40	6	0.7406	0.7750	5.9	40	6	0.7138	0.7250	5.7
Bru09	36	15	0.7118	0.7778	13.5	40	13	0.8153	0.8250	11.8	40	14	0.7525	0.7500	12.7
Ssosl417	36	10	0.8488	0.8056	9.7	40	10	0.8109	0.8250	9.2	40	10	0.8338	0.8250	9.5
Ssosl438	36	4	0.3738	0.3333	4.0	40	4	0.4628	0.4750	3.9	40	4	0.5916	0.6000	3.9
Str73	36	3	0.5112	0.4722	2.8	40	3	0.5150	0.5000	3.0	40	3	0.5209	0.4250	3.0
Average	35.7	8.7	0.6393	0.6500	8.2	39.8	8.4	0.6525	0.6673	7.8	39.8	8.2	0.6475	0.6494	7.6

Loci	L (*N* = 324)					ALL (*N* = 550)				
	*N*	*N*_all_	*H*_e_	*H*_o_	*A*_r_	*N*	*N*_all_	*H*_e_	*H*_o_	*A*_r_
Strutta12	317	24	0.8999	0.8580	13.8	540	25	0.8954	0.8463*	14.0
Str15	319	7	0.7669	0.7179	5.9	544	7	0.7690	0.7482	5.8
Strutta58	322	18	0.8865	0.8571	12.9	544	21	0.8907	0.8695	13.2
Str60	323	4	0.3637	0.3591	2.3	549	4	0.3813	0.3625	2.2
Bru13	321	20	0.8397	0.8131	12.3	541	21	0.8509	0.8373	12.2
Bru14	323	6	0.3680	0.3375	4.3	549	6	0.3663	0.3515	4.2
Bru22	322	2	0.2337	0.2391	2.0	542	2	0.2594	0.2546	2.0
Bru25	322	31	0.8916	0.8323*	16.2	548	34	0.8983	0.8467*	16.6
Bru07	324	8	0.7909	0.7500	6.2	550	9	0.7877	0.7727	6.3
Bru09	323	25	0.7253	0.6780	11.9	541	35	0.7286	0.6969	12.8
Ssosl417	322	10	0.8143	0.8075	8.6	548	11	0.8267	0.8139	8.9
Ssosl438	322	5	0.4728	0.4627	3.9	547	5	0.4610	0.4388	3.9
Str73	322	3	0.5556	0.4938	3.0	548	3	0.5532	0.5055	3.0
Average	321.7	12.5	0.6622	0.6312*	7.9	545.5	14.1	0.6668	0.6419*	8.1

Significant departure from HW (*p* < .05) after sequential Bonferroni correction is marked “*”.

**Table A2:** Weir and Cockerham's (1984) pairwise *F*_ST_ (θ) estimate among trout from riverine sites (R) and lake caught trout >35 cm (L) assigned to riverine sites in GENECLASS2

Site/Assigned site (*n*)	RP1	RP2	RP3	RT1	RT2	RT3	LP1	LP2	LP3	LT1	LT2	LT3
RP1 (40)		0.0201	0.0549	0.0228	0.0341	0.0305	0.0039	0.0189	0.0444	0.0261	0.0302	0.0305
RP2 (40)	**		0.0618	0.0125	0.0224	0.0250	0.0224	−0.0045	0.0435	0.0210	0.0170	0.0255
RP3 (30)	**	**		0.0394	0.0651	0.0626	0.0580	0.0565	0.0088	0.0379	0.0520	0.0511
RT1 (36)	**	**	**		0.0157	0.0176	0.0204	0.0128	0.0205	−0.0023	0.0107	0.0139
RT2 (40)	**	**	**	**		0.0132	0.0310	0.0238	0.0514	0.0306	0.0046	0.0243
RT3 (40)	**	**	**	**	**		0.0254	0.0272	0.0434	0.0277	0.0200	0.0048
LP1 (32)	*ns*	**	**	**	**	**		0.0178	0.0410	0.0183	0.0201	0.0179
LP2 (45)	**	*ns*	**	**	**	**	**		0.0369	0.0151	0.0141	0.0196
LP3 (36)	**	**	*ns*	**	**	**	**	**		0.0154	0.0308	0.0306
LT1 (54)	**	**	**	*ns*	**	**	**	**	**		0.0149	0.0103
LT2 (15)	**	**	**	*	*ns*	**	**	**	**	**		0.0144
LT3 (18)	**	**	**	*	**	*ns*	**	**	**	*	*ns*	

Nonsignificant (*ns*) and significant test (*, **, ***) after sequential Bonferroni correction according to 5%, 1%, and 0.1%, respectively.
